# Transcriptome Analysis Identifies a Gene Cluster for the Biosynthesis of Biruloquinone, a Rare Phenanthraquinone, in a Lichen-Forming Fungus *Cladonia macilenta*

**DOI:** 10.3390/jof7050398

**Published:** 2021-05-20

**Authors:** Wonyong Kim, Min-Hye Jeong, Sung-Hwan Yun, Jae-Seoun Hur

**Affiliations:** 1 Korean Lichen Research Institute, Sunchon National University, Suncheon 57922, Korea; minhye1962@gmail.com; 2 Department of Medical Sciences, Soonchunhyang University, Asan 31538, Korea; sy14@sch.ac

**Keywords:** biruloquinone, *Cladonia*, polyketide, PKS, secondary metabolite

## Abstract

Lichens are prolific producers of natural products of polyketide origin. We previously described a culture of lichen-forming fungus (LFF) *Cladonia macilenta* that produces biruloquinone, a purple pigment that is a phenanthraquinone rarely found in nature. However, there was no genetic information on the biosynthesis of biruloquinone. To identify a biosynthetic gene cluster for biruloquinone, we mined polyketide synthase (PKS) genes from the genome sequence of a LFF isolated from thalli of *C. macilenta*. The 38 PKS in *C. macilenta* are highly diverse, many of which form phylogenetic clades with PKS previously characterized in non-lichenized fungi. We compared transcriptional profiles of the 38 PKS genes in two chemotypic variants, one producing biruloquinone and the other producing no appreciable metabolite in vitro. We identified a PKS gene (hereafter PKS21) that was highly upregulated in the LFF that produces biruloquinone. The boundaries of a putative biruloquinone gene cluster were demarcated by co-expression patterns of six clustered genes, including the PKS21. Biruloquinone gene clusters exhibited a high degree of synteny between related species. In this study we identified a novel PKS family responsible for the biosynthesis of biruloquinone through whole-transcriptome analysis.

## 1. Introduction

More than a thousand polyketide-derived secondary metabolites (SMs) have been isolated from lichen thalli, many of which are exclusively found in lichens [1,2,3]. Several polyketide-derived SMs, such as barbatic acid, didymic acid, rhodocladonic acid, and thamnolic acid, have been extracted from lichen thalli of *Cladonia macilenta* Hoffm. [4,5]. In addition, a polyketide, biruloquinone, was discovered in an isolated mycobiont of *Cladonia macilenta* [6]. Biruloquinone is a phenanthraquinone rarely found in nature. It was first discovered in lichen thalli of *Parmelia birulae* [7], and later in a plant pathogenic fungus *Mycosphaerella rubella* [8]. Lichen-forming fungi (LFF) occasionally produce novel phenolic compounds that have not been found in lichen thalli grown in nature [9]. Many of these compounds derived from lichen thalli or LFF culture exhibit interesting biological activities, including anti-oxidative, antibacterial, anti-inflammatory, antifungal, and anticancer properties [3,9,10]. Biruloquinone also has pharmaceutical potential, inhibiting acetylcholinesterase, one of the key enzymes involved in Alzheimer’s disease progression [6].

Fungi produce structurally diverse polyketides, such as the health-threatening aflatoxin [11], the cholesterol-lowering drug lovastatin [12,13], and melanin pigments [14,15,16,17] via iterative type I polyketide synthase (PKS). Fungal PKS can be broadly classified into non-reducing PKS (NR-PKS), partially reducing (PR-PKS) and highly reducing PKS (HR-PKS), based on PKS domain architecture. In addition, there are PKS–nonribosomal peptide synthetase (NRPS) hybrid enzymes that comprise PKS domains found in PR-PKS and a module of NRPS. The ketoacyl synthase (KS) domain of PKS has been considered evolutionarily conserved, and thus it has served as a proxy for the similarity of the entire PKS [18]. Given the highly oxygenated tetracyclic core of biruloquinone, it was predicted to be biosynthesized by an NR-PKS [19]. Thioesterase (TE) domains of NR-PKS have been functionally diverged to have different product release mechanisms that may have led to structural diversity of polyketide-derived compounds. Polyketide product release mechanisms by TE domains could be generally differentiated into five distinct modes [20]: (i) macrolactone formation (intramolecular cyclization via O–C bond formation), (ii) depside or melleolide formation (intermolecular cross-coupling via O–C bond formation), (iii) hydrolytic release, (iv) pyrone formation (intramolecular cyclization via O–C bond formation), and (v) Claisen condensation (intramolecular cyclization via C–C bond formation, followed by deacetylation in some cases).

**Figure 1:**
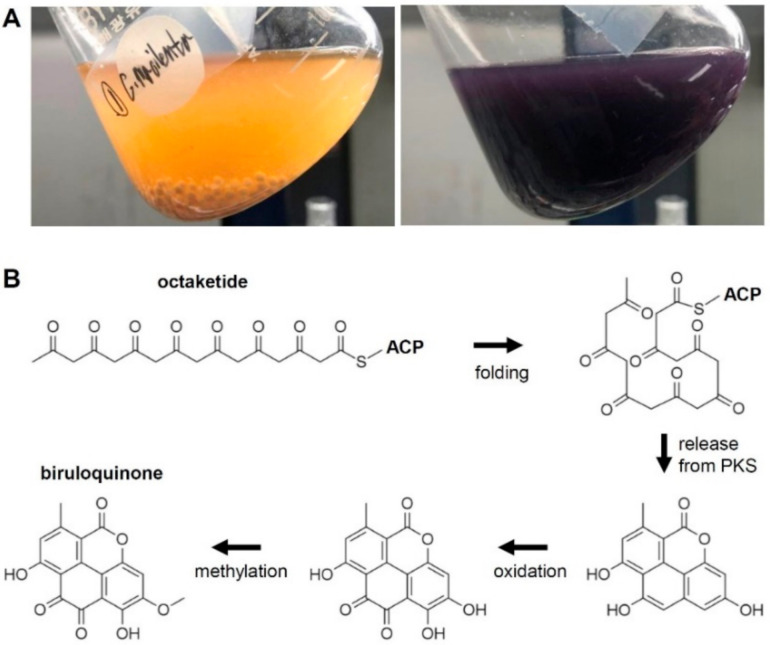
Production of biruloquinone. (**A**) Accumulation of biruloquinone (purple pigment) in an axenic culture of *Cladonia macilenta* LFF (right panel). Left panel shows an axenic culture of *C. macilenta* LFF devoid of biruloquinone. (**B**) A proposed polyketide pathway for the biosynthesis of biruloquinone.

In our previous study, LFF were isolated from thalli of *C. macilenta* [19]. Among the isolated LFF, two distinct chemotypes were observed: some produced a copious amount of purple pigment, which was structurally identified as biruloquinone [6], and the others produced no such pigment in axenic culture (Figure 1A; Supplementary Figure S1). To investigate genome-encoded metabolic potentials of *C. macilenta*, we sequenced the genome of one of the isolated LFF with purple pigmentation [21]. The aims of this study were to explore metabolic potentials of *C. macilenta* by categorizing PKS and to identify a biosynthetic gene cluster (BGC) responsible for the production of biruloquinone through whole-transcriptome analysis.

## 2. Materials and Methods

### 2.1. Genome Annotation and Biosynthetic Gene Cluster Identification

The genome assembly of *C. macilenta* [21] was annotated using the GenSAS (v.6.0) annotation pipeline [22]. Default settings were used unless otherwise noted. In brief, low-complexity regions and repeats were masked using RepeatModeler (v1.0.11) and RepeatMasker (v4.0.7) (www.repeatmasker.org, accessed on 5 February 2020), setting the DNA source to ‘Fungi’. A masked consensus sequence was generated, on which ab initio gene prediction was performed using the following tools: (i) Augustus (v3.3.1) [23], selecting *As. nidulans* as a trained organism; (ii) GeneMark-ES (v4.33) [24]; (iii) Genscan (v1.0) [25], using a parameter setting for Human and other vertebrates; and (iv) GlimmerM (v2.5.1) [26], selecting *Aspergillus* as a trained organism. For homology-based predictions, the NCBI reference transcript and protein databases for Fungi were searched, using (v) BLAST+ (v2.7.1) [27] and (vi) DIAMOND (v0.9.22) [28], respectively. For the consensus gene model prediction using EVidenceModeler (v06-25-2012) [29], the above-mentioned standalone gene predictions were weighted as follows: (i) five, (ii) ten, (iii) one, (iv) one, (v) five, and (vi) five. A total of 10,705 open reading frames were predicted in the current genome annotation. For genome mining, the genome assembly and annotation files of *C. macilenta* were processed by the antiSMASH program (v5.0+) [30], with a parameter setting: ‘--minimal’.

### 2.2. Phylogenetic Analysis

KS domain sequences were extracted from 38 PKS (19 NR-PKS and 19 R-PKS) identified in the *C. macilenta* genome sequence, and 111 PKS (62 NR-PKS and 49 R-PKS) that have been linked to known compounds in other filamentous fungi (Supplementary Table S1), using the online tool NaPDoS [31]. A total of 149 KS domain sequences were aligned using MAFFT (v7.310) [32] with the ‘auto’ setting, and the resulting multiple sequence alignment was trimmed for poorly aligned regions using Gblocks (v0.91b) [33] with the parameter: ‘-b4 = 5’. A maximum likelihood tree was constructed using the RAxML program (v8.2) [34] and annotated by iTOL (v5.7) [35]. Nodal supports were evaluated by 1000 bootstrap replications.

### 2.3. RNA-Seq Experiment

LFF isolated from thalli of *C. macilenta* (accession number: KoLRI003786) were obtained from the Korean Lichen and Allied Bioresource Center at Sunchon National University (Suncheon, Korea). A LFF producing biruloquinone (purple strain, accession number KoLRI021765) and a LFF producing no appreciable metabolite (white strain, accession number KoLRI021766) were cultured on malt extract agar media (BD Biosciences, Baltimore, MD, USA) for revitalization. The colonies of purple and white strains were harvested and homogenized to be mycelial solutions in sterilized water, using a bead beater. Approximately 100 mg of shredded mycelia were inoculated into malt extract broth (MEB) (BD Biosciences, Baltimore, MD, USA). The purple and white strains were grown in 100 mL of MEB at 18 °C in an orbital shaker (150 rpm). Fifteen days after incubation, production of biruloquinone was confirmed in the purple strain by a high-performance liquid chromatography (HPLC) analysis (Supplementary Figure S1). Chromatographic separation was achieved on a Prominence Modular LC-20A HPLC instrument (Shimadzu, Kyoto, Japan), as previously described [19]. After chemical profiling, mycelia were harvested, ground to a fine powder in liquid nitrogen, and subjected to total RNA extraction using an easy-spin total RNA extraction kit (iNtRON Biotechnology, Seoul, Korea). cDNA libraries were constructed, using the TruSeq RNA library preparation kit (San Diego, CA, USA), and sequenced on the HiSeq2000 platform at Macrogen Inc. (Seoul, Korea). The quality of raw reads (paired-end, 100 bp) was assessed with the FastQC program (v0.11.3; www.bioinformatics.babraham.ac.uk/projects/fastqc, accessed on 10 November 2019). Then, Illumina adapters were trimmed, and poor-quality reads with a mean quality lower than 15 in 4-bp sliding windows from 3′ ends of reads were trimmed, and trimmed reads shorter than 36 bp were further filtered out using the Trimmomatic program [36]. Filtered reads were mapped to the genome sequence of *C. macilenta* using the HISAT2 program (v2.1.0) [37]. Gene expression levels in reads per kilobase per million mapped reads (RPKM) values were computed and normalized by effective library size estimated by trimmed mean of M values, using the edgeR R package (v3.26.8) [38].

### 2.4. Identification of Syntenic Gene Clusters

Genome assemblies of *Cladonia* species were downloaded from the Joint Genome Institute (JGI) or the National Center for Biotechnology Information (NCBI): *C. borealis* (NCBI accession: JAFEKC000000000), *C. grayi* (JGI accession: Cgr/DA2myc/ss v2.0), *C. metacorallifera* (NCBI accession: GCA_000482085.2), *C. rangiferina* (NCBI accession: GCA_006146055.1), and *C. uncialis* (NCBI accession: GCA_002927785.1). The genome assemblies were annotated using the GenSAS pipeline, and BGCs were mined from the five related *Cladonia* species, using antiSMASH, as described above. To search for homologous BGCs in the six *Cladonia* spp., we used the BiG-SCAPE program [39] that is useful for investigating the conservation and variation of BGCs in related species. Based on the Jaccard index of domain types, domain sequence similarity, and domain adjacency index, the BiG-SCAPE program calculates a similarity matrix between pairwise combinations of clusters where smaller values indicate greater BGC similarity [39]. A cutoff value of 0.5 was used to identify homologous gene clusters.

### 2.5. Data Availability

The sequences of the biruloquinone BGC and its flanking genes in *C. macilenta* (Cma_00864–Cma_00876) have been submitted to GenBank and may be found under accession number MW980728–MW980740. The RNA-seq data for transcriptome analysis of *C. macilenta* LFF have been deposited in NCBI SRA (PRJNA723447).

## 3. Results

### 3.1. The Biosynthesis of Biruloquinone

On the basis of the chemical structure, biruloquinone is proposed to be biosynthesized from an octaketide that is likely biosynthesized by an NR-PKS (Figure 1B) [19]. A proposed folding mechanism of an octaketide product in the catalytic cavity of a yet-unknown NR-PKS suggested that a pyrone ring can be formed when the polyketide intermediate is released by a hydrolytic activity of the TE domain of NR-PKSs, as in the case of the cercosporin biosynthetic pathway [40]. The resulting tetracyclic aromatic ring needs to be further modified by tailoring enzymes to yield biruloquinone. The most parsimonious routes of biruloquinone formation require oxidation of two carbon units and methylation of a hydroxyl group (Figure 1B).

### 3.2. Phylogenetic Placement of C. macilenta PKS

**Figure 2:**
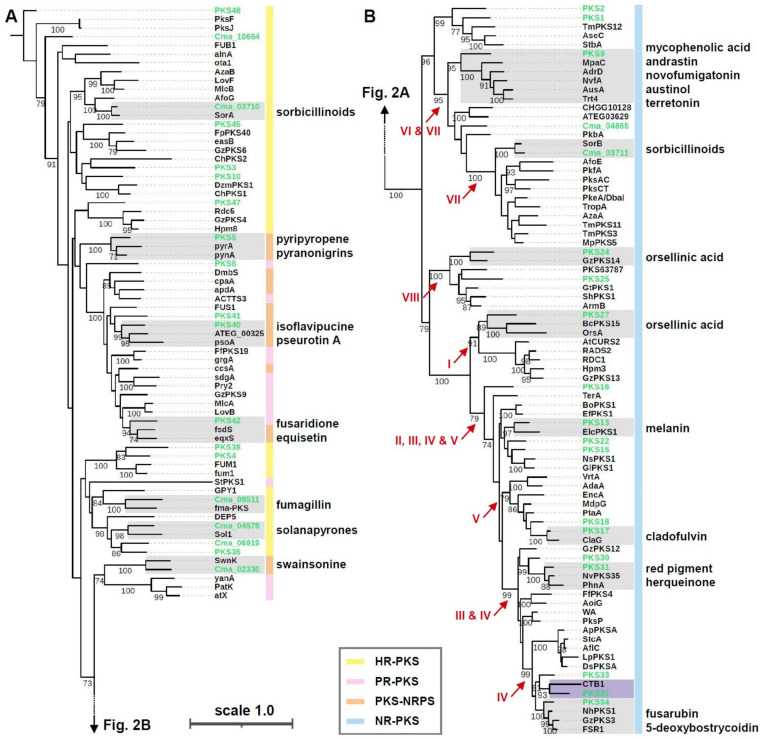
Genealogy of fungal PKS. (**A**) Phylogeny of reducing type (R)-PKS, including highly reducing (HR)-PKS, partially reducing (PR)-PKS, and PKS-non-ribosomal peptide synthetase (NRPS) hybrid enzyme. (**B**) Phylogeny of non-reducing type (NR)-PKS. Roman numerals in red indicate eight phylogenetic groups of fungal NR-PKS. Shaded boxes indicate phylogenetic clades (bootstrap values greater than 90) including a *C. macilenta* PKS and one or more PKSs characterized in other filamentous fungi. End products biosynthesized by PKSs in other filamentous fungi are denoted in the phylogenetic clades (see Supplementary Table S1). The numbers at the internal nodes indicate bootstrap values greater than 70 from 1000 bootstrap replications. Branch lengths are proportional to the inferred amount of evolutionary change, and the scale represents 1.0 amino acid sequence substitutions per site.

To search for candidate PKSs responsible for the biosynthesis of biruloquinone, we mined the genome of *C. macilenta*, using antiSMASH. A total of 38 PKS genes were identified: 19 NR-PKSs and 19 R-PKSs (Supplementary Table S1). Since PKS genes are similar in their deduced amino acid sequences, and PKS domain architecture tends to afford end products similar in chemical structure, we analyzed the 38 *C. macilenta* PKS in an evolutionary framework, with 62 NR-PKSs and 49 R-PKSs that have been characterized in other filamentous fungi (Supplementary Table S1). Phylogenetic analysis of KS domain sequences revealed two major PKS clades: R-PKS (Figure 2A) and NR-PKS (Figure 2B). The R-PKS clade includes three different PKS types differing in PKS domain architecture: HR-PKS, PR-PKS, and PKS-NRPS hybrid enzymes (Figure 2A). The NR-PKS clade has been grouped into seven [41] or eight major groups [42] by protein sequence similarity and PKS domain architecture. We also identified the eight previously described groups of NR-PKS (I–VIII) (Figure 2B). The PKS genes identified in *C. macilenta* are highly diverse, forming phylogenetic clades with many different PKSs previously characterized in non-lichenized fungi (Figure 2). For candidate PKSs for the biosynthesis of biruloquinone, we focused on the NR-PKS groups II, III, IV, and V. NR-PKSs in these groups are known to afford polycyclic compounds, such as aflatoxin and melanin, while the NR-PKS groups I, VI, VII, and VIII are known to produce monocyclic compounds, such as orsellinic acid [41,42]. In *C. macilenta*, eleven PKSs (PKS13, PKS15, PKS16, PKS17, PKS18, PKS21, PKS22, PKS30, PKS31, PKS33, PKS34) were predicted to be involved in biosynthesis of polycyclic compounds.

### 3.3. Transcriptome Analysis of C. macilenta PKS

**Figure 3:**
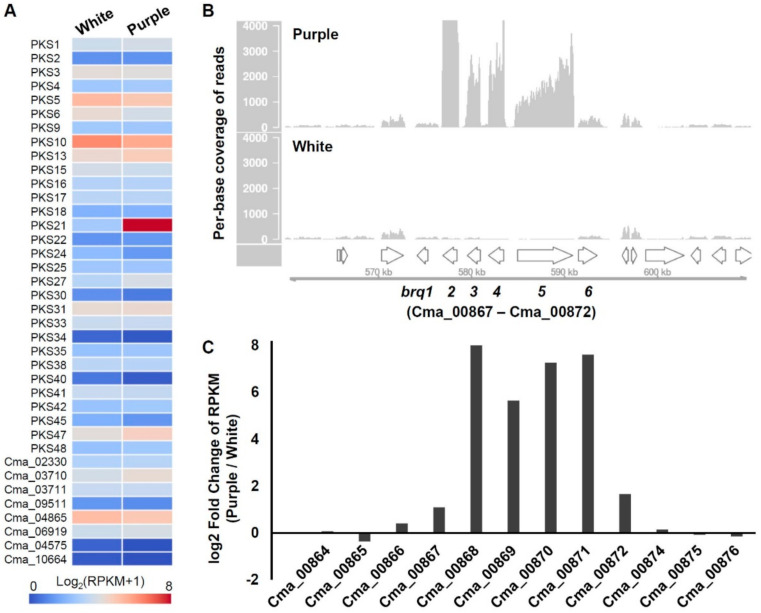
Identification of biruloquinone PKS and associated biosynthetic gene cluster. (**A**) Expression values (Log_2_-transformed (RPKM+1)) are shown as heat maps for 38 PKS genes in a LFF that lacks biruloquinone production (white) and a LFF that produces biruloquinone (purple) in axenic culture. (**B**) Per-base coverage of RNA-seq reads was plotted for a genomic locus surrounding the PKS21 (*brq5*; gene ID: Cma_00871). RNA-seq reads mapped on the *C. macilenta* reference genome were subsampled to 60 million reads for visual comparison of expression levels between white (lower panel) and purple (upper panel). Arrows on the *x*-axis indicate genes *(brq1*–*brg6*) and flanking genes. The numbers on the *y*-axis are per-base coverage. (**C**) Log2-transformed fold change of RPKM values in purple compared to white. RPKM, reads per kilobase per million mapped reads.

To narrow down candidate PKS genes responsible for the biosynthesis of biruloquinone, we performed RNA-seq experiments and compared PKS gene expression levels between the purple strain and the white strain in axenic culture [19]. Among the 38 PKS genes, we observed that an NR-PKS (PKS21) was highly upregulated in the purple strain, while the other PKS genes exhibited similar expression levels in the purple and white strains (Figure 3A). The expression of the PKS21 was 195-fold greater in the purple strain, suggesting that the PKS21 is likely involved in the biosynthesis of biruloquinone. The PKS21 belongs to the NR-PKS group IV and is most closely related to CTB1 (Figure 2B), an NR-PKS involved in the biosynthesis of cercosporin in *Cercospora nicotianae* [40]. The TE domain of CTB1 is shown to directly catalyze pyrone formation during the ring cyclization process of the cercosporin intermediate [40]. A pyrone formation is also required for the biosynthesis of the biruloquinone intermediate when it is released from PKS enzyme (Figure 1B). The close phylogenetic relationship between CTB1 and the PKS21 further strengthened the putative assignment of the PKS21 to the biosynthesis of biruloquinone.

### 3.4. Identification of Biruloquinone Biosynthesis Gene Clusters

As the biosynthesis of biruloquinone requires *O*-methylation at a hydroxyl group and oxidation at two different carbon units (Figure 1B), we examined flanking regions of the PKS21 gene to search for related biosynthetic enzymes. Expectedly, we found two genes, encoding *O*-methyltransferase (gene ID: Cma_00869) and FAD-dependent monooxygenase (Cma_00870) (Table 1), which may be required for tailoring the biruloquinone intermediate. In addition, there were two major facilitator superfamily (MFS) genes (Cma_00868 and Cma_00872) and a gene encoding a GAL4-type transcription factor (Cma_00867), surrounding the PKS21 gene (Table 1). To determine the borders of a biruloquinone BGC in *C. macilenta*, we compared expression levels of genes neighboring the PKS21 between the purple strain and white strain. Visualization of RNA-seq reads mapped on the genome of *C. macilenta* indicated that the six genes related to secondary metabolism (*brq1*–*brg6*; Table 1) were coordinately upregulated in the purple strain, while the six genes remained nearly silent in the white strain (Figure 3B). Expression levels of other genes of hypothetical function flanking the six genes (*brq1*–*brg6*) were basal and similar between the purple and white strains (Figure 3B,C). Taken together, we were able to delimit the borders of the biruloquinone BGC in *C. macilenta*.

**Figure 4:**
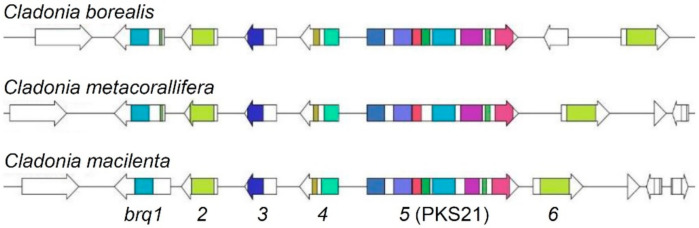
Synteny of biruloquinone BGCs in *Cladonia* species. Arrows indicate open reading frames (ORFs) found in biruloquinone BGC (*brq1*–*brg6*) loci in the three *Cladonia* species. Pfam domains related to secondary metabolism are color-coded in predicted ORFs (see Table 1).

To investigate whether other *Cladonia* species also possess a homologous BGC for the biosynthesis of biruloquinone, we mined genome sequences of five *Cladonia* species, *C. borealis*, *C. grayi*, *C. metacorallifera*, *C. rangiferina*, and *C. uncialis*. The BiG-SCAPE program [39] identified a homologous BGC in *C. borealis* and *C. metacorallifera*. However, the genomes of *Cladonia grayi*, *C. rangiferina*, and *C. uncialis* did not carry a homologous BGC. The biruloquinone BGCs identified in *C. borealis* and *C. metacorallifera* were highly syntenic to the one in *C. macilenta*, including all the six core genes (*brq1*–*brg6*) (Figure 4), suggesting that *C. borealis* and *C. metacorallifera* also have genetic potential to produce biruloquinone.

## 4. Discussion

Despite huge genome-encoded metabolic potentials of lichen-forming fungi [43,44], little is known about biosynthetic genes responsible for lichen metabolite production. Ascribing specific biosynthetic genes to lichen metabolites has been slow, due to a paucity of genetic information and lack of molecular tools for manipulating LFF recalcitrant to genetic transformation. To circumvent these issues, positive correlations between expression levels of biosynthetic genes and production of metabolites of interest have been used as circumstantial evidence for linking biosynthetic genes to lichen metabolites [45,46,47].

We previously described CmaPKS1 as a candidate PKS for the biosynthesis of biruloquinone by analyzing the phylogenetic relationship and PKS domain architecture of CmaPKS1 [19]. After our reclassification of *Cladonia* PKS genes, CmaPKS1 was henceforth renamed PKS15, forming a phylogenetic clade with GlPKS1 and NsPKS1 that are known to be involved in melanin production in non-lichenized fungi [16,17]. Thus, this putative assignment was erroneous, and, in this study, we provided layers of evidence that the PKS21 (*brq5*) is responsible for the biosynthesis of biruloquinone: (i) the PKS21 was the sole PKS upregulated in the LFF-producing biruloquinone; (ii) the PKS21 was closely-related to CTB1 that directly catalyzes pyrone formation, an activity required for the biosynthesis of biruloquinone and cercosporin; and (iii) the putative biruloquinone gene cluster (*brq1*–*brg6*) included tailoring enzymes required for the biosynthesis of biruloquinone, such as *O*-methyltransferase and FAD-dependent monooxygenase.

The boundaries of the putative biruloquinone gene cluster were determined by the co-expression of six genes (*brq1*–*brg6*). The gene cluster included two MFS-type transporter genes, *brq2* and *brq6*. The *brq2* exhibited a dramatic increase (256-fold) in its expression level in the LFF-producing biruloquinone, while the expression of the *brq6* exhibited only three-fold greater in the LFF-producing biruloquinone. Although the precise function of the two transporters remains to be investigated, the *brq2* may play a major role in pumping the purple pigment, biruloquinone, out to culture media. Fungal biosynthetic gene clusters typically harbor one or more pathway-specific transcription factors that regulate clustered genes in a coordinated manner [48]. The gene cluster also included a gene encoding GAL4-type transcription factor (*brq1*) that can be found in many biosynthetic gene clusters in filamentous fungi. However, the *brq1* lacks a DNA-binding domain and contains only the middle homology region commonly found in typical Zn(II)2Cys6 zinc cluster TFs, such as yeast GAL4. This might be due to misannotation of the *brq1* in our current genome assembly, but there was a report that a GAL4-type TF only with the middle homology region regulates a fungal biosynthetic gene cluster [49]. The *brq1* exhibited only two-fold increase in expression in the purple strain, and this slight upregulation seems to be sufficient to induce the other clustered genes in the purple strain.

It is currently unknown how chemotypic variation is introduced to produce biruloquinone in *C. macilenta* LFF isolated from the same lichen thallus. Production of unexpected secondary metabolites (not observed in natural lichens) in axenic culture of LFF have been frequently observed [6,50,51,52,53]. These chemotypic variations may be attributable to phenotypic instability of isolated mycobionts. A significant proportion of biosynthetic gene clusters in fungi remain silenced in normal growth conditions and are subject to tight controls by epigenetic regulators (e.g., histone modifiers) [54,55,56,57,58,59,60,61,62,63]. Thus, it is conceivable that the biruloquinone BGC was derepressed by dysfunctionally regulated histone modifiers due to genomic instability of isolated mycobionts, which otherwise remains silenced during the symbiosis.

In this study, we connected a biosynthetic gene cluster to biruloquinone with high likelihood. Homologous biruloquinone BGCs were also found in related *Cladonia* species (subsection *Erythrocarpae* [64]). Given the observations that biruloquinone is produced by different fungal taxa, such as *C. fruticulosa* (subclade *Graciles* in the genus *Cladonia*), *Parmelia birulae,* and *Mycosphaerella rubella* [7,8,65], production of rare phenanthraquinone-derived metabolites is more widespread than previously thought. Ascribing the PKS21 (*brq5*) to biruloquinone helps elucidate the evolutionary origin of this unique PKS family in fungi.

## Figures and Tables

**Table 1:** The biruloquinone biosynthetic gene cluster in *Cladonia macilenta*.

ORF ^a^ (Cma)	Size (aa)	BLASTP Homolog (Accession)	% Identity (Coverage)	Conserved Domain	*E*-Value
00864	325	extradiol dioxygenase (XP_037155234)	71 (100)	Memo-like protein (Pfam01875)	6 × 10^−77^
00865	393	hypothetical protein (CAF9912135)	26 (31)	(not detected)	-
00866	737	hypothetical protein (CAF9931462)	60 (96)	(not detected)	-
**00867**	699	GAL4-type transcription factor (PMD28964), *brq1*	56 (80)	fungal-specific transcription factor (Pfam04082)	1 × 10^−14^
**00868**	452	MFS transporter (OCK96312), *brq2*	75 (99)	Major Facilitator Superfamily (Pfam07690)	1 × 10^−21^
**00869**	425	*O*-methyltransferase (KAA6412585), *brq3*	74 (99)	*O*-methyltransferase (Pfam00891)	4 × 10^−14^
**00870**	430	FAD-dependent monooxygenase (KAA6409270), *brq4*	72 (99)	NAD(P)-binding Rossmann-like domain (Pfam13450)	2 × 10^−7^
**00871**	1971	polyketide synthase (QIX11496), *brq5*	94 (100)	ketoacyl synthase (Pfam00109)	8 × 10^−88^
**00872**	590	MFS transporter (KAA6410135), *brq6*	71 (96)	Major Facilitator Superfamily (Pfam07690)	3 × 10^−32^
00873	167	hypothetical protein (XP_018191177)	30 (60)	(not detected)	-
00874	191	hypothetical protein (CAF9930055)	86 (100)	CS domain (Pfam04969)	5 × 10^−19^
00875	159	hypothetical protein (XP_037155314)	87 (98)	Polysaccharide biosynthesis (Pfam04669)	7 × 10^−38^
00876	970	ABC efflux pump (SLM35520)	50 (99)	ABC transporter transmembrane region (Pfam00664)	3 × 10^−35^

^a^ ORF: open reading frames; genes for the biruloquinone biosynthetic gene cluster are highlighted in bold.

## Data Availability

Not applicable.

## Supplementary Materials

The following are available online at https://www.mdpi.com/article/10.3390/jof7050398/s1, Supplementary Figure S1: Detection of biruloquinone in lichen-forming fungi (LFF) isolated from *Cladonia macilenta*, Supplementary Table S1: Known PKS characterized in non-lichenized fungi and PKS identified in *Cladonia macilenta.*
