# Supplementary material for: Transcriptome Analysis Identifies a Gene Cluster for the Biosynthesis of Biruloquinone, a Rare Phenanthraquinone, in a Lichen-Forming Fungus Cladonia macilenta
Source: J Fungi (Basel). 2021 May 20;7(5):398. doi: 10.3390/jof7050398 (PMC8161216; doi:10.3390/jof7050398)
Supplement: Supplementary file 1 [file jof-07-00398-s001.zip › Supplementary_Figure_S1.pdf]

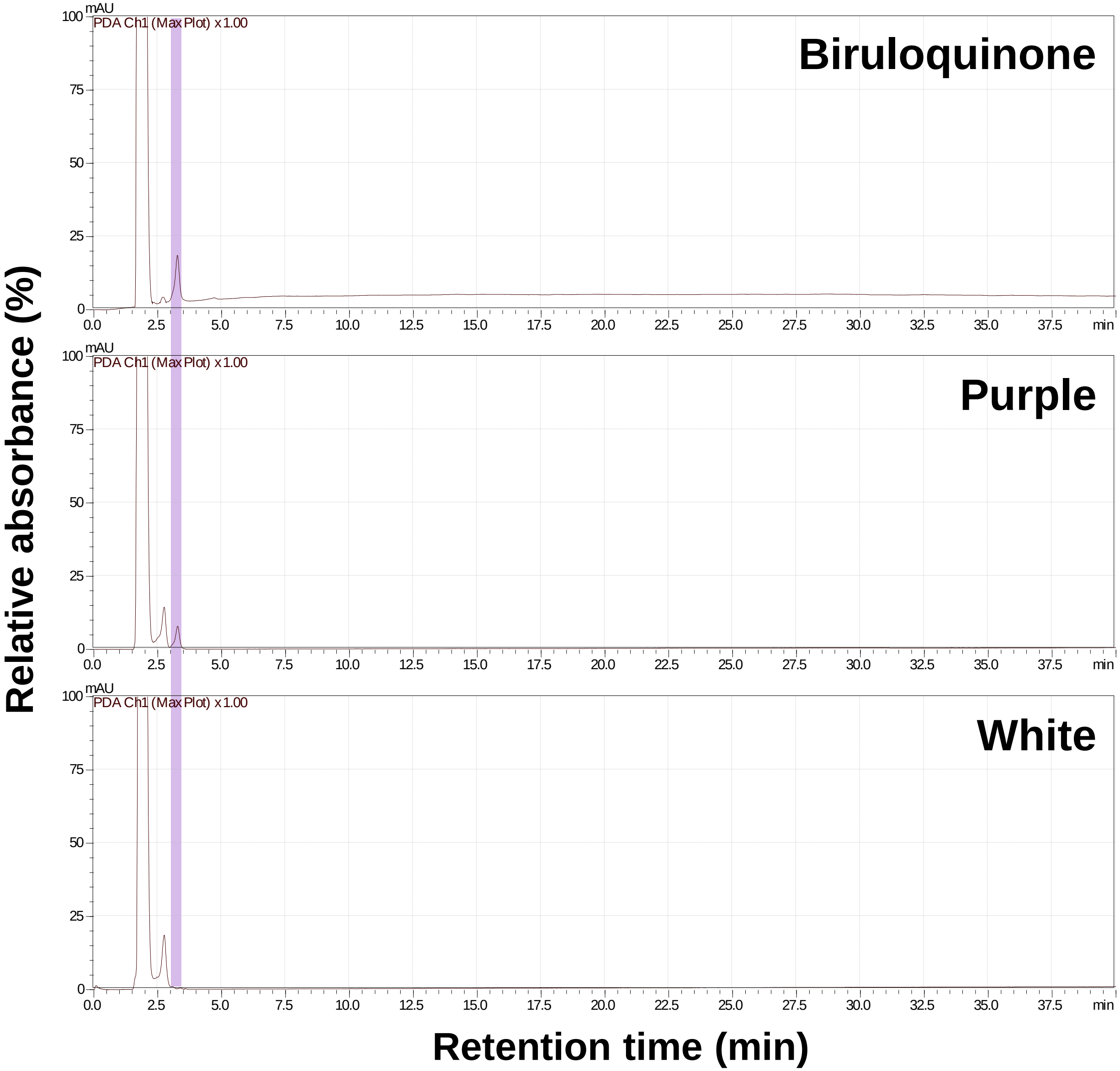

Supplementary Figure S1. Detection of biruloquinone in lichen-forming fungi (LFF) isolated from *Cladonia macilenta*. HPLC profiles of acetone extracts of LFF culture of the purple strain (middle panel) and the white strain (lower panel). Upper panel is the chromatogram of purified biruloquinone (t_R = 3.26 min) dissolved in acetone (t_R = 1.85 min).
